# Homeotic transformations reflect departure from the mammalian ‘rule of seven’ cervical vertebrae in sloths: inferences on the *Hox* code and morphological modularity of the mammalian neck

**DOI:** 10.1186/s12862-018-1202-5

**Published:** 2018-06-07

**Authors:** Christine Böhmer, Eli Amson, Patrick Arnold, Anneke H. van Heteren, John A. Nyakatura

**Affiliations:** 1 UMR 7179 CNRS/MNHN, Muséum National d’Histoire Naturelle, 57 rue Cuvier, CP-55 Paris, France; 2 AG Morphologie und Formengeschichte, Institut für Biologie, Humboldt Universität zu Berlin, Philippstraße 13, 10115 Berlin, Germany; 3 Image Knowledge Gestaltung: An Interdisciplinary Laboratory, Humboldt University, Philippstraße 13, 10115 Berlin, Germany; 4 Museum für Naturkunde, Leibniz-Institut für Evolutions- und Biodiversitätsforschung, Invalidenstraße 43, 10115 Berlin, Germany; 5 Institut für Zoologie und Evolutionsforschung mit Phyletischem Museum, Ernst-Haeckel-Haus und Biologiedidaktik, Friedrich-Schiller-Universität Jena, Erbertstraße 1, 07743 Jena, Germany; 6 Department of Human Evolution, Max Planck Institute for Evolutionary Anthropology, Deutscher Platz 6, 04103 Leipzig, Germany; 7 Sektion Mammalogie, SNSB - Zoologische Staatssammlung, Münchhausenstraße 21, 81247 München, Germany; 8 GeoBio-Center, Ludwig-Maximilians-Universität München, Richard-Wagner-Straße 10, 80333 Munich, Germany; 9 Department Biologie II, Ludwig-Maximilians-Universität München, Großhaderner Straße 2, 82152 Planegg-Martinsried, Germany

**Keywords:** Axial patterning, *Hox* genes, Evolution, Constraint, Xenarthra, Cervical vertebrae

## Abstract

**Background:**

Sloths are one of only two exceptions to the mammalian ‘rule of seven’ vertebrae in the neck. As a striking case of breaking the evolutionary constraint, the explanation for the exceptional number of cervical vertebrae in sloths is still under debate. Two diverging hypotheses, both ultimately linked to the low metabolic rate of sloths, have been proposed: hypothesis 1 involves morphological transformation of vertebrae due to changes in the *Hox* gene expression pattern and hypothesis 2 assumes that the *Hox* gene expression pattern is not altered and the identity of the vertebrae is not changed. Direct evidence supporting either hypothesis would involve knowledge of the vertebral *Hox* code in sloths, but the realization of such studies is extremely limited. Here, on the basis of the previously established correlation between anterior *Hox* gene expression and the quantifiable vertebral shape, we present the morphological regionalization of the neck in three different species of sloths with aberrant cervical count providing indirect insight into the vertebral *Hox* code.

**Results:**

Shape differences within the cervical vertebral column suggest a mouse-like *Hox* code in the neck of sloths. We infer an anterior shift of *HoxC-6* expression in association with the first thoracic vertebra in short-necked sloths with decreased cervical count, and a posterior shift of *HoxC-5* and *HoxC-6* expression in long-necked sloths with increased cervical count.

**Conclusion:**

Although only future developmental analyses in non-model organisms, such as sloths, will yield direct evidence for the evolutionary mechanism responsible for the aberrant number of cervical vertebrae, our observations lend support to hypothesis 1 indicating that the number of modules is retained but their boundaries are displaced. Our approach based on quantified morphological differences also provides a reliable basis for further research including fossil taxa such as extinct ‘ground sloths’ in order to trace the pattern and the underlying genetic mechanisms in the evolution of the vertebral column in mammals.

**Electronic supplementary material:**

The online version of this article (10.1186/s12862-018-1202-5) contains supplementary material, which is available to authorized users.

## Background

The mammalian ‘rule of seven’ vertebrae in the neck is a striking case of evolutionary stasis [1–3]. In contrast to non-mammalian amniotes, mammals are highly constrained in the number of cervical vertebrae (CV) and their neck kinematics rely on interspecific variation in vertebral morphology, but not in vertebral count [4, 5]. The developmental origin of this meristic constraint in the mammalian neck, however, is still under debate [2, 6, 7]. One explanation posits that numerical changes in cervical count may be coupled with indirect selection due to pleiotropy (e.g., [8–10]). Pleiotropy refers to the phenomenon of mutations in a single gene that affect more than one phenotypic character of an organism [10–12]. Changes in pleiotropic genes were found to be associated with effects that dramatically lower fitness (malformations and cancers), and thus may indirectly constrain evolutionary change through stabilizing selection (e.g., [8, 11, 13]). Indeed, skeletons of mammals with an atypical number of CV show many anomalies, such as fusion of vertebrae, defective production of cartilage, abnormal ossification of sternum and pelvic girdle, abnormal fibrous bands connected to rudimentary ribs, and asymmetric ribs [2, 14, 15]. A further explanation states that the meristic constraint arose as a byproduct of the origin of the muscularization of the mammalian diaphragm [6, 7]. The migrating muscle precursor (MMP) cells that form the diaphragm originate from the midcervical somites and migrate posteriorly [6]. Both an anterior and posterior transposition of the forelimb (resulting in less or more cervicals, respectively) are likely to generate a deficiency in the development of the muscularized diaphragm, of the forelimb, or both [6]. This specialization of the midcervical somites associated with the MMP cells responsible for the developing mammalian diaphragm became again subject to stabilizing selection after its first appearance in the Triassic [7].

Recent work revealed a correlation between anterior *Hox* gene expression and the quantifiable shape of the CV in living archosaurs [16]. Although differing in the number of cervical vertebrae, birds and crocodiles share a common underlying *Hox* gene modularity. Following this, changes in the expression of the underlying genetic code can be hypothesized solely from quantifiable vertebral morphology (e.g., for fossils with known cervical morphology, (see [16])). A similar correlation and modularity was subsequently shown for the mammalian model species mouse [17], the only mammal for which the *Hox* gene expression patterns have been established [18, 19]. Three morphological subunits were detected in the postatlantal cervical vertebral column of the mouse indicating that two distinct shape changes occur between successive CV (between C2 and C3 and between C5 and C6) [17]. This morphological modularity detected in the mouse appears to represent the general pattern for living mammals with seven CV [6, 17, 20] with overall cervical spine length as the main source of variation in the mammalian neck [21]. A conservative complex of C3–5 responsible for the development of the muscularized diaphragm [6] is in line with this observed modularity in the mouse. Although phylogenetically diverse, there is evidence for a common *Hox* code in living placental mammals as they appear to display similar patterns of morphological differentiation within the neck, which is thought to reflect a common developmental regionalization [6, 22]. It was suggested that this pattern might even be valid for all synapsids (with seven CV) [17]. In order to have a similar validation as in archosaurs, however, morphological-developmental patterns in mammals with different numbers of vertebrae have to be examined. Differences in the number of vertebrae with cervical identity would imply a morphological regionalization of the neck that corresponds to modifications in *Hox* gene expression domains (expansion of a *Hox* gene’s expression domain and/or a shift of gene expression) [16, 23].

Besides manatees (Sirenia), sloths (Xenarthra) are the only mammals departing from the mammalian ‘rule of seven’ vertebrae in the neck. They display either reduced or increased cervical counts and show considerable intraspecific variation in the number of CV [1–3, 15, 24–27]. Consequently, analyses of these exceptions were conducted to further our understanding of the evolutionary conservatism in the mammalian axial skeleton which is also the objective of the present study. The two living, distantly related genera of sloths (belonging to the Megalonychidae and Bradypodidae) are superficially similar (folivorous, arboreal, upside-down posture, suspensory locomotion) [28], but two-toed sloths (*Choloepus*) have five to eight CV while three-toed sloths (*Bradypus*) have eight to ten CV [3, 15, 26, 29, 30]. *Choloepus* usually has a relatively short and robust neck and keeps its head upside-down during suspensory postures. *Bradypus*, in contrast, has a remarkably long and flexible neck and is reported to be able to rotate its head for 270° in both directions [31, 32]. Like the developmental origin of the mammalian ‘rule of seven’ CV (see above), the explanation for the exceptional number of CV in sloths is still under debate [2, 15, 24–27], yet a mechanism that allows sloths to depart from this ‘rule’ is clearly involved.

**Fig. 1:**
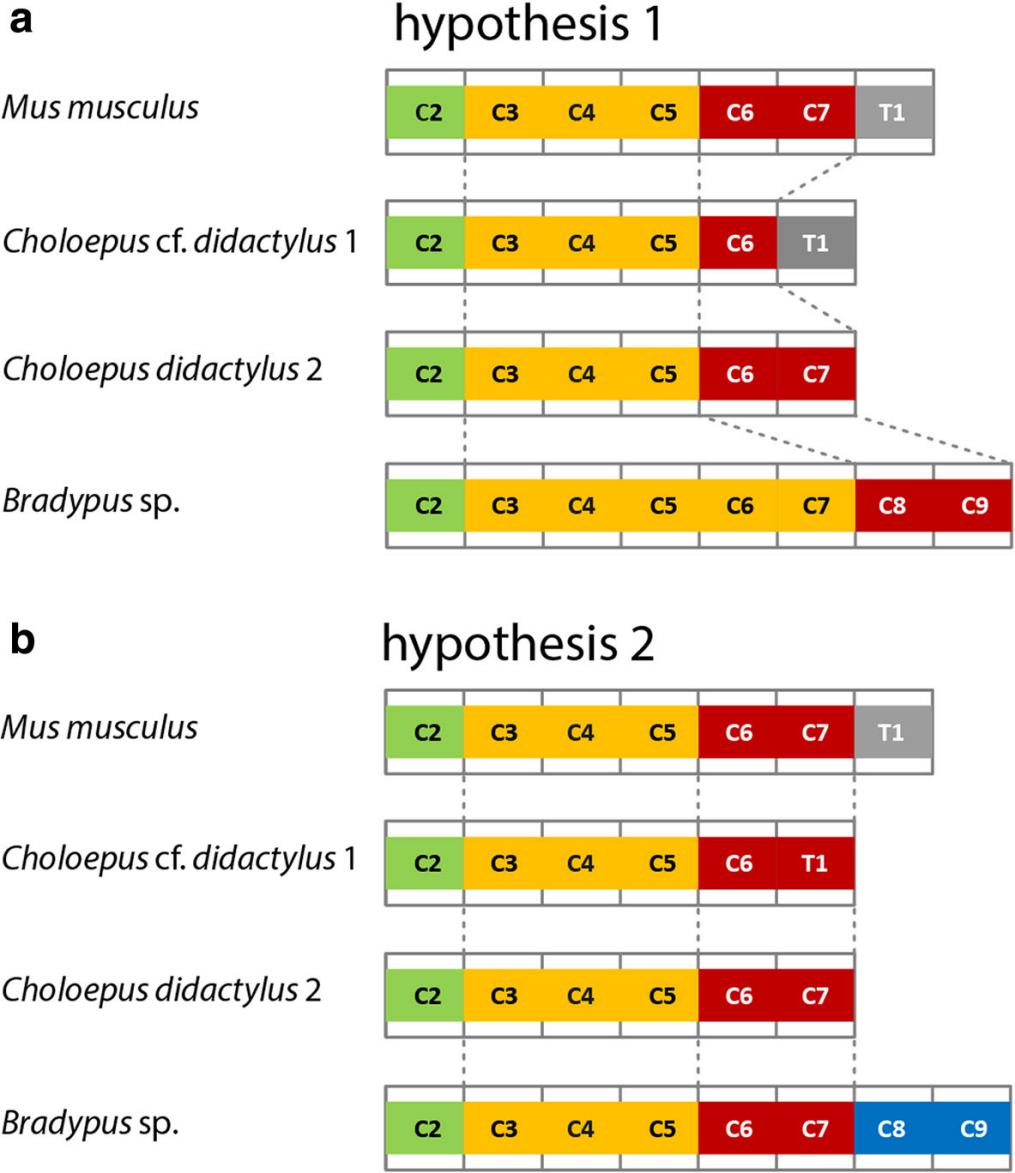
Two evolutionary hypotheses of mechanisms to break the seven cervical vertebrae (CV) constraint in sloths. The *Hox* code is a key determinant of vertebral identity and the color coding represents modules with the same *Hox* code. A three-subunit pattern within the postatlantal CV has been found (green, axis; yellow, anterior; red, posterior). Thoracic vertebrae shown in grey. A: The first hypothesis predicts that the number of CV is changed due to an altered *Hox* code. Therefore, the modular pattern in the neck of sloths with an aberrant number of CV should differ from that of living mammals (represented by the mouse), e.g., due to expansion of one of the subunits. B: The second hypothesis predicts that the first seven vertebrae retain a cervical identity and the *Hox* code remains unchanged. According to this hypothesis the modular pattern in sloths corresponds to the general pattern of living mammals and CV that are originally thoracic vertebrae are added (blue)

There are two hypotheses for the breaking of the meristic constraint underlying the aberrant vertebral count in the sloths’ neck compared to the previously described mouse pattern: (1) The number of modules is retained but their boundaries are displaced and (2) the number of modules is altered but their boundaries are retained (Fig. 1). For both hypotheses, seemingly diverging but not mutually exclusive evidences from development have been provided (see below). The first hypothesis assumes changes in the *Hox* gene expression pattern that result in the morphological transformation of vertebrae (i.e., homeosis) [1, 2, 14, 15]. A shorter neck, according to the homeosis hypothesis, is associated with homeotic transformation of CV into thoracic vertebrae, whereas a longer neck is the result of homeotic transformation of thoracic vertebrae into CV. Despite the pleiotropic effects described above, low metabolic rates in sloths were proposed to result in relaxed selection regimes [2, 8, 15, 33]. The concept of homeosis, however, is here not applied in the strict definition of Bateson [30], which implies local shifts of individual serial elements while the others remain unchanged (e.g., C7 anatomy is replaced by thoracic vertebra 1 anatomy in *Choloepus* specimens with six CV). We rather understand homeosis here as global developmental changes sensu Buchholtz & Wayrynen (2014) [34]. These global changes involve displacements of boundaries between axial regions but with the full range of cervical anatomies still present and thus a dissociation of axial patterning and segmentation [34].

The second hypothesis assumes that the *Hox* gene expression pattern is not altered and the identity of the vertebrae is not changed [7, 26, 27, 31]. This involves a level of independence of components of the axial skeleton derived from somitic vs. those derived from lateral plate mesoderm (primaxial/abaxial repatterning, PAR). Again, this does not exclude a role for stabilizing selection [26, 27]. Accordingly, the number of CV and the modular boundaries remain the same, but a shift of the abaxial elements (pectoral girdle and limbs) occurred relative to the adjacent and stationary primaxial domain (vertebrae) resulting in shorter and longer necks, respectively. Indeed, the vertebral ossification pattern indicates that the posterior CV in the long-necked sloths (*Bradypus*) are developmentally thoracic [27]. Importantly, this second hypothesis, as the first one, assumes that the extremely low metabolic rates of sloths allow for their apparent tolerance to unusual cervical counts [7]. Furthermore, both hypotheses are consistent with a diaphragm-linked origin of the cervical constraint [7].

The present study takes advantage of the recent work that revealed the correlation between anterior *Hox* gene expression patterns and the quantifiable shape of the CV [16, 17] to gain insight into the aberrant cervical count in sloths. We used three-dimensional (3D) geometric morphometrics combined with a relative warps analysis (see methods) to quantify shape differences between species (interspecific dataset) and within the cervical series of each specimen (intraspecific dataset) separately. Since hypothesis 1 predicts that the number of vertebrae with cervical identity is changed, whereas hypothesis 2 predicts that the first seven vertebrae retain cervical identity, we here test (1) whether the morphological pattern of sloths with seven CV corresponds to the postulated general modularity pattern of living mammals (i.e., the three-subunit pattern); (2) whether the morphological pattern of sloths with more or less than seven CV indicates a change in the number of vertebrae with cervical identity; and (3) whether the alteration in the number of CV is accompanied by a displacement of boundaries between modules. This will help to further elucidate the evolutionary mechanism behind the conservatism of cervical count in mammals and its departure in sloths.

## Results

**Fig. 2:**
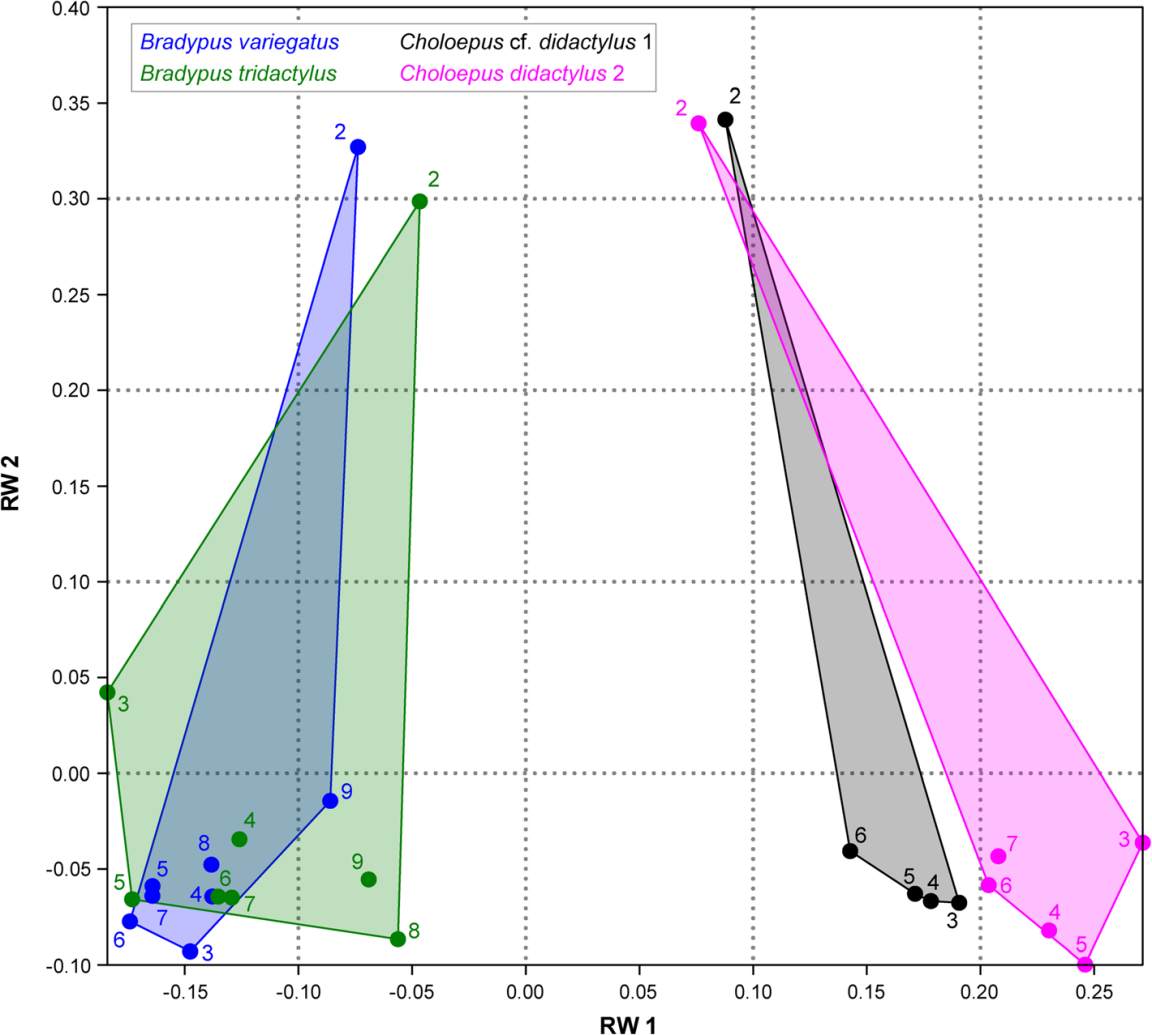
Relative warps (RW) analysis results of the interspecific dataset. The plot shows that the two genera *Choloepus* and *Bradypus* occupy distinct regions of the morphospace. *B. variegatus* and *B. tridactylus* cluster together, whereas the two specimens, *C. didactylus* do so as well but show less overlap. In all analyzed sloths, the cervical vertebra 2 is very distinct in its morphology and is separate from the postaxial vertebrae

The landmark-based 3D geometric morphometric analysis of the interspecific data set (including all CV of the four specimens together) showed that *Choloepus* and *Bradypus* occupy distinct regions of the morphospace (Fig. 2). *Bradypus variegatus* and *B. tridactylus* cluster together, whereas the *C.* cf. *didactylus* 1 and *C. didactylus* 2 do so as well, but show less overlap. In all analyzed sloths, C2 is very distinct in its morphology and is separate from the postaxial vertebrae.

**Fig. 3:**
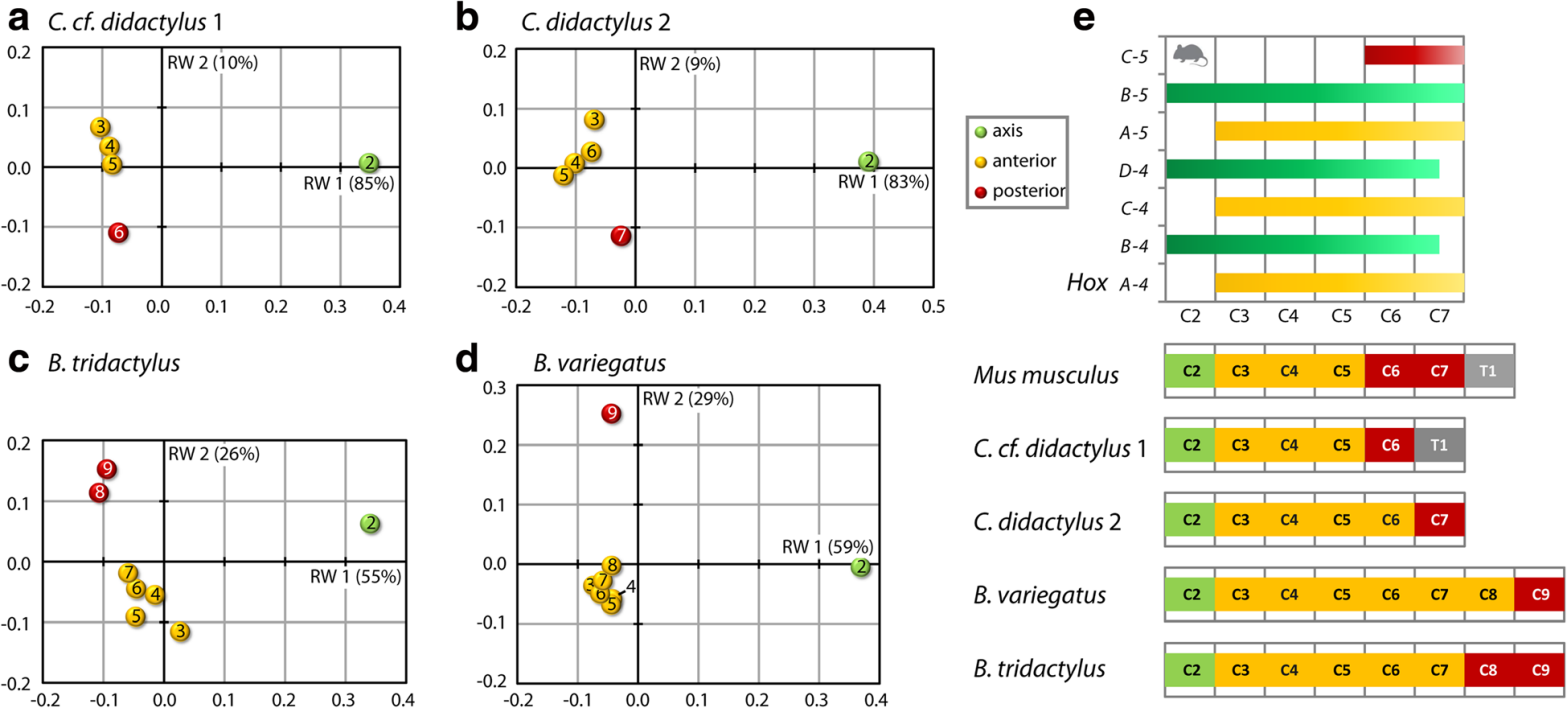
Relative warps analysis results of the intraneck dataset. The plots show the shape differences within the cervical vertebral column along relative warp (RW) 1 and RW 2 for each specimen (A-D). The morphological analysis allowed discrimination of the vertebrae in three different subunits (indicated by color coding) (E). The correlation between somitic *Hox* gene expression pattern and morphological modularity in the neck of the mouse (*Mus musculus*) is based on Böhmer et al. (2015b) [23] and Böhmer (2017) [17]. *Hox4 and Hox5* genes are expressed in the cervical vertebral column of the mammal. The anterior expression limit of *HoxC-6* (not shown in the figure) lies at the first thoracic vertebra (T1). Corresponding to the mouse, two distinct shape changes are revealed between successive CV in all analyzed sloths. The modular pattern in the neck of sloths with additional CV differs in displaying an expanded anterior region of the neck (yellow)

The intraneck data set (treating each specimen separately) revealed a distinct morphological differentiation of the neck in all analyzed sloths (Fig. 3a-d). At least 80% of the total variance in the sample is explained by the first two relative warps (Additional file 1: Table S1) and, thus, the morphospace constructed from relative warp 1 and relative warp 2 provides a reasonable approximation of the total shape variation. Relative warp 1 separates C2 from the postaxial vertebrae in each taxon whereas relative warp 2 separates the postaxial vertebrae into an anterior and a posterior group (Fig. 3). The posterior group comprises the last cervical vertebra in *C.* cf. *didactylus* 1, *C. didactylus* 2, and *B. variegatus*, but the last two CV in *B. tridactylus*.

**Fig. 4:**
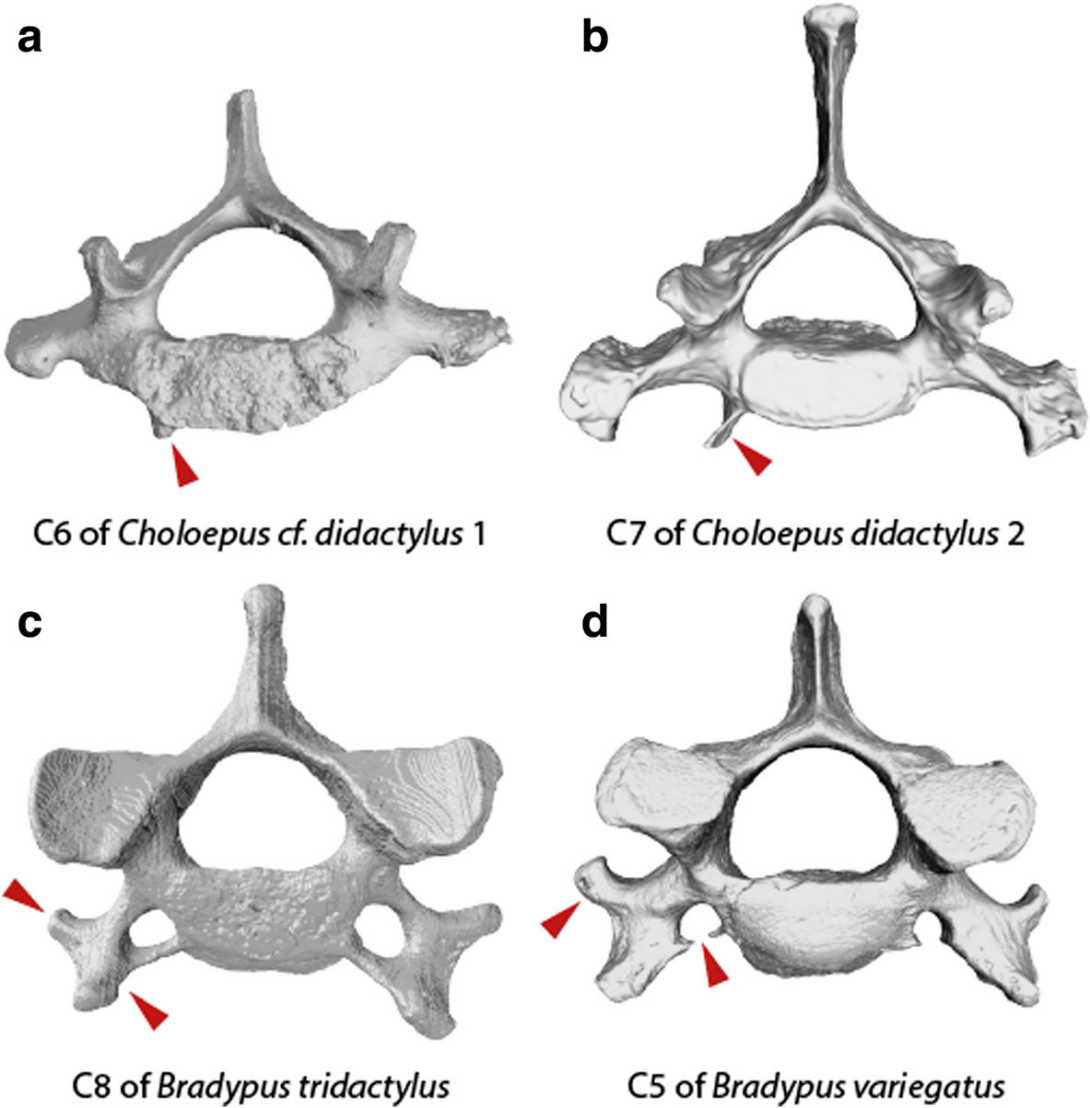
Congenital anomalies were found in all analysed sloth specimens. Anterior view of different, 3D rendered CV with red arrow heads pointing to malformations. A, the last cervical vertebra (C6) of *C.* cf. *didactylus* 1 displays unilaterally a ventral process. B, the last cervical vertebra (C7) of *C. didactylus* 2 shows unilaterally a ventral process. C, lateral transverse processes of C7 and C8 in *B. tridactylus* are not developed symmetrically (C8 shown here). D, on the C5 in *B. variegatus* the foramina transversaria of are not entirely closed and transverse processes are not developed symmetrically

As confirmed by the cluster analysis (Additional file 1: Figure S1), the relative warps analysis allowed discrimination of the vertebrae. In all sloths analyzed, the geometric morphometric analysis revealed a three-subunit pattern (Fig. 3e). The common modular pattern comprises the axis (C2), an anterior, and a posterior unit. The distribution of the CV onto the specific modules, however, reveals variation among species. In *C.* cf. *didactylus* 1 (six CV), the modular pattern includes C2, three anterior (C3–5), and one posterior (C6) vertebrae. Including the first thoracic vertebra (V7 in this specimen) in the morphometric analyses confirmed that it is very distinct in its morphology (not shown here), as it does not cluster with C6. In *C. didactylus* 2 (seven CV), the morphological subunits comprise C2, four anterior CV (C3–6), and one posterior (C7) cervical vertebra. The morphological subunits of both long-necked sloths (nine CV), *B. variegatus* and *B. tridactylus,* comprised C2, six anterior CV (C3–8) and one posterior (C9) cervical vertebra. Examples of congenital anomalies were found in all specimens (Fig. 4, Additional file 1: Figure S2).

## Discussion

### Qualitative vs. quantitative morphology

The modular pattern in sloths as quantified by the present morphological analysis is in agreement with the extensive qualitative survey of vertebral morphology by Varela-Lasheras et al. (2011) [15]. The first five or six CV of *Choloepus* and the first eight CV of *Bradypus* have an unambiguous cervical shape [15], which corresponds to the atlas, axis, and the anterior morphological subunit (this study). The last vertebra (C6, 7, and 9, respectively) in the neck always had a transient cervicothoracic shape and rudimentary ribs [15], which corresponds to the posterior morphological subunit (this study). Furthermore, Varela-Lasheras et al. (2011) [15] observed that the penultimate vertebra in the neck of *Bradypus* (C8) had a transitional cervicothoracic shape in two specimens. Indeed, the modular pattern of *B. variegatus* and *B. tridactylus* differs in our study in regard of the assignment of C8 to the anterior or posterior morphological subunit (Fig. 3c, d). A study of morphological modularity in the vertebral column of modern cats (Felidae) correspondingly revealed a transitional module comprising the last two CV and the first two thoracic vertebrae (C6-T2) [35].

### Sloths with seven CV and the postulated general pattern of living mammals

The morphological three-subunit pattern found in the neck of the mouse is viewed as representing the general pattern for living mammals with seven CV [6, 17, 20]. Two distinct shape changes occur in the post-atlantal cervical series between successive CV: between C2–3 and between C5–6 [17]. The present study detected a corresponding modular, three-subunit pattern for *C. didactylus* 2 (seven CV), but the distinct shape change in the posterior part of the neck occurs between C6–7 rather than between C5–6. On the basis of the correlation between anterior *Hox* gene expression and quantifiable vertebral shape of the CV in the mouse, this could possibly be the result of the expression of *HoxC-5* being shifted posteriorly by one vertebra in *C. didactylus* 2. Thus, the number of modules in the neck of the sloth with seven CV is the same as for the mouse, but the boundary between the anterior and posterior module is shifted.

Morphological specialization to the suspensory lifestyle of sloths may contribute to the difference in their modular pattern. The CV form a mobile, multi-jointed structure with complex kinematics and requiring the coordination of many muscles [36–38]. A reduction of this complexity could be achieved by the regionalization of the cervical vertebral column [20, 39–44]. The functionally specialized vertebrae form three compartments and such a reduced geometry was suggested to facilitate motor control of the neck [39, 40]. Normally, the cervicothoracic transition involves the last two CV (C6–7), but it appears to be reduced in sloths involving only the last vertebra in the neck. This may again be linked to morpho-functional aspects of their cervical vertebral column. It has been shown in the giraffe that the attachment area of a neck flexor muscle (*Musculus longus colli*) is shifted posteriorly, which was interpreted as providing more flexibility to the neck [45]. Suggesting a similar close relationship for the axial musculoskeletal system of sloths, the highly specialized locomotor mode of *Choloepus* is reflected in the intramuscular architecture of their dorsovertebral muscles [46, 47]. In terms of shoulder muscles, the development of the serratus ventralis and the rhomboideus may be linked to the posterior subunit in the neck of sloths. Parts of both muscles originate from the posterior CV and insert on the scapula [48, 49]. In general, the main function of these muscles is suspension of the thorax between the forelimbs. Due to the inverse body orientation in sloths, however, they are not suited to fulfill this role [48]. Correspondingly, these muscles are weakly developed or even absent [32, 48, 50]. This may entail that the posterior subunit comprises only the last vertebra instead of the last two CV as in the mouse.

### Sloths with a decreased number of CV

The modular pattern in the cervical vertebral column of *C.* cf. *didactylus* 1 (six CV) corresponds to the pattern detected in the neck of *C. didactylus* 2 (seven CV), but is shifted by one vertebra since the neck is shorter. Thus, the hypothesis 1 (same number of modules, displacement of module boundaries) is favored. The distinct shape change in the posterior part of the cervical vertebral column occurs between the two last vertebrae (C5–6). This suggests that the anterior expression limit of *HoxC-5* is similar to that of the mouse at C6, but the seventh vertebra appears to be homeotically transformed into a thoracic vertebra in the sloth. However, since the complete range of vertebral morphologies is present (Fig. 4; C6 resembles C7 in *C. didactylus* 2), the homeotic transformation is global rather than local (see [34]). More specifically, this indicates that the anterior expression limit of *HoxC-6* is shifted anteriorly by one vertebra in *C.* cf. *didactylus* 1. It has been shown that the expression of the *HoxC-6* gene starts at the first thoracic vertebra in a variety of amniotes that differ in cervical count [19, 23, 51]. It corresponds to the transition from cervical to thoracic vertebrae (i.e., cervicothoracic transition) in the mouse (seven CV), chicken (14 CV), goose (17 CV), crocodile (nine CV) and turtle (eight CV) [19, 23, 51]. A similar modular pattern as observed here in the cervical vertebral column of *Choloepus* has been reported for manatees (genus *Trichechus*) [34]. The reduction in cervical count in *Trichechus* from seven to six resembles a similar global homeotic shift based on a global dissociation of the processes of somitogenesis and axial patterning [34]. It thus further highlights the global validity of the modular pattern of the neck observed here.

Congenital anomalies (e.g., asymmetric vertebrae or vertebrae with rudimentary ribs) were found in all specimens including *C.* cf. *didactylus* 1 (Fig. 4a, Additional file 1: Figure S2) and have also previously been reported [15, 26]. Such transitional vertebral identities may be the result of *Hox* gene mutations and pleiotropic effects and hint at homeotic transformations [15]. However, such morphologies have also been argued to be consistent with shifts between primaxially (i.e., of somitic origin) and abaxially (i.e., originating from lateral plate mesoderm) derived tissues in line with the primaxial/abaxial repatterning (PAR) hypothesis [26]. It is also important to point out, in this context, that the patterning of the mesoderm plate is not collinear [52].

### Sloths with an increased number of CV

The two *Bradypus* specimens studied here had more than seven CV, but still displayed a morphological three-subunit pattern in the neck corresponding to the general mammalian pattern. This results in a large anterior morphological subunit comprising five vertebrae in one individual and six vertebrae in the other individual (Fig. 3e). Correlated with the relative position of the posterior subunit, this indicates again that the number of modules is retained but the boundaries between them are displaced (hypothesis 1). The displacement is likely based on a posterior shift of *HoxC-5* and *HoxC-6* expression in the long-necked sloths. The difference in the assignment of C8 to the anterior (in *B. variegatus*) or posterior (in *B. tridactylus*) morphological subunit may suggest either a functional difference or interspecific variation. Since both species do not distinctly differ in behavior and are phylogenetically very close [53, 54], it seems more likely that this part of the neck is subject to interspecific variation.

The last two CV (C8–9) in long-necked sloths are the only vertebrae in the neck whose centra ossify before other cervical centra and neural arches [27]. This observation was taken as evidence supporting an underlying PAR because the early ossification of C8–9 resembles the anterior-most rib-bearing thoracic vertebrae of other mammals [27] (note that this inference was also critized [15]). Therefore, the number of vertebrae with a cervical identity was interpreted as unchanged in *Bradypus* and it was inferred that no changes occurred in the *Hox* code [27]. Although the complete range of vertebral morphologies is ‘stretched’ to eight or nine vertebrae, respectively (Fig. 3), it is not possible to exclude this possibility without any further genetic data. However, a complex, integrated network of signaling pathways and gene regulators governs bone formation and these molecular mechanisms are not independent of *Hox* gene activity [55].

### Cervical ribs and *HoxA-5*

In contrast to vertebrae, which develop solely from the somitic mesoderm, the ribs derive from both somitic and lateral plate mesoderm [52, 56, 57]. Only the distalmost part of the rib (sternal rib) depends on the lateral plate mesoderm and is considered to be an abaxial element [52, 56, 57]. The rib head, neck, tubercle, and proximal part of the body of the rib derive from the somitic mesoderm and are considered axial elements [56, 57]. Following the PAR hypothesis, this suggests a shift between vertebrae including the proximal part of the ribs (primaxial structures) and the distalmost part of the ribs (abaxial structures) [26]. Although the present results indicate homeotic transformations of the vertebrae in sloths with aberrant number of CV, the PAR hypothesis cannot be fully rejected in short-necked sloths since the sternal ribs are indeed shifted anteriorly. For long-necked sloths, the PAR hypothesis appears not to be supported because the increase in the number of CV is also associated with the loss of cervical ribs. However, considering that the increase in cervical count is also associated with a shift of the pelvis [26], none of the hypotheses can explain axial anatomy in *Bradypus* alone and parallel processes might be involved.

Several studies reported on the association between the development of cervical ribs and the expression of *HoxA-5* in amniotes [16, 17, 23, 51, 52, 58–60]. Alligators and crocodiles possess free cervical ribs and the expression limit of *HoxA-5* starts in the posterior cervical vertebral column [16, 23, 58]. In birds, whose cervical ribs are present, but fused to the vertebrae, the anterior expression limit of *HoxA-5* is in the middle region of the neck [58] and *HoxA-5* knockdown results in defects of the cervical ribs [61]. This contrasts with the more anterior *HoxA-5* expression observed in the mouse [58]. The loss of free ribs on the CV (by reduction and fusion) in Mammaliaformes during the Cretaceous [6, 61–63], suggests that the anterior expression limit of *HoxA-5* is shifted, hence resembling the pattern of the mouse [17]. Since the CV in sloths often bear irregular fused riblets, it may be possible that *HoxA-5* expression starts in the posterior region of the neck. It is not likely that a shift of the anterior expression limit of *HoxA-5* occurred twice within one lineage, hence this may indicate that either the lineage towards modern sloths retained a posterior *HoxA-5* expression, or the shift is a side-effect of the change in the expression of other *Hox* genes, in particular *HoxC-5* and *HoxC-6*. Future studies are required to further elucidate the evolutionary role of *HoxA-5* in axial patterning. Furthermore, the determination of the *Hox* gene inventory for sloths by PCR (polymerase chain reaction) survey would be a first crucial step in order to validate for the presence of *Hox* genes in comparison with other mammals.

### Is the mammalian ‘rule of seven’ actually a ‘rule of three’?

Our morphological findings of the CV of sloths and the inferred *Hox* gene expression patterns support the hypothesis that the number of modules in the neck is retained but the borders between the modules are displaced when the cervical count is altered (hypothesis 1). Similar results were obtained for *Trichechus*, the only other mammalian genus with an aberrant number of CV [34]. Thus, the general validity of the three-subunit pattern of the mouse [17] is confirmed even under varying vertebral number. However, the underlying developmental basis for the meristic variation in sloths cannot be clarified by our morphological approach as neither global homeotic changes nor PAR alone can explain all aspects of sloth skeletal anatomy. It is likely that different processes act in *Choloepus* and *Bradypus* or, as they are not mutually exclusive (see above), that parallel processes shape the overall anatomy of sloths necks (see [34]). Nevertheless, it becomes evident that the mammalian neck is not constrained to seven CV per se, but to the conserved modularity of underlying *Hox* gene expression dividing the neck into three subunits. As most amniote lineages, synapsids increased the number of cervical vertebrae from six (basic amniote pattern) to seven in therapsids resulting in increased head mobility [64]. Further meristic variation, however, was prevented by strong developmental constraints based on the conserved *Hox* gene pattern since the Jurassic [2, 6]. Thus, the limitation to the number of seven CV is a byproduct of the arrested increase of cervical count. Nevertheless, variation in cervical number is not completely repressed as demonstrated by the sloths (and also the manatee). The three-subunit pattern, in contrast, seems not to be changeable even for those aberrant mammals.

## Conclusion

Remarkably, sloths display variation in the number of CV, but a three-subunit morphological pattern in the neck is conserved - a pattern that appears to be common for mammals in general. In contrast to archosaurs (cf. [16, 23]), the correlation between anterior *Hox* gene expression and quantifiable shape of the CV found in sloths with aberrant cervical count further indicates that the *Hox* code may be conserved across living mammals. Our results are consistent with a mouse-like *Hox* code in the cervical vertebral column of sloths, with an anterior shift of *HoxC-6* expression in association with the first thoracic vertebra in short-necked sloths, and a posterior shift of *HoxC-5* and *HoxC-6* expression in long-necked sloths. In combination with the presence of vertebral anomalies, these observations provide insights into the homeotic processes during development that involve morphological transformation of vertebrae as a result of changes in the *Hox* gene expression pattern. Nevertheless, the occurrence of PAR and thus related shifts of abaxial elements cannot be rejected by our findings. As both hypotheses may not be mutually exclusive, it is possible that parallel processes shape the overall anatomy of sloths necks [34]. Moreover, processes resulting in the variation of cervical count are not necessarily the same in both genera. In conclusion, since hypothesis 1 (number of modules is retained but their boundaries are displaced) was not rejected by our analysis but was instead favored by the morphological modularity found here, both explanations remain plausible and may both be involved in the mammalian constraint of cervical count: indirect selection due to pleiotropy [2] and a developmental constraint associated with the muscular diaphragm in mammals [6].

Future analyses on the *Hox* code in non-model organisms, such as sloths, may yield direct evidence for the evolutionary mechanism responsible for the morphological adaptability of the axial skeleton. However, to date, the realization of these analyses is extremely limited. The present study provides a basis for further research that should include extinct taxa in order to trace the pattern and the underlying genetic mechanisms in the evolution of the vertebral column. Ultimately, this will further our understanding of the processes responsible for the 200-million-year old evolutionary constraint and the conserved three-subunit pattern that shapes mammalian cervical evolution [62].

### Methods

The present study includes the complete cervical vertebral column of four sloths with variable number of vertebrae in the neck comprising three different species (Table 1).

**Table 1:** Specimens analyzed in the present study (ZMB, Museum für Naturkunde Berlin, Germany)

Taxon	# Cervical vertebrae	Collection number
*Choloepus* cf. *didactylus* 1	6	ZMB_MAM_102634
*Choloepus didactylus* 2	7	ZMB_MAM_38388
*Bradypus variegatus*	9	ZMB_MAM_35824
*Bradypus tridactylus*	9	ZMB_MAM_76147

### Quantitative morphological analysis

The morphological variability within the cervical vertebral column is evaluated by geometric morphometrics. A linear regression method for landmark-based geometric morphometrics of vertebrae has been described by Head & Polly (2015) [65], but requires a minimum of at least 10–20 observations for regression analysis (cf. [66]). Therefore, we here follow the procedure applied by Böhmer et al. (2015a) [16] which allows the statistical assessment of shape changes between successive vertebrae in vertebral series comprising less than 10 vertebrae.

Our approach allows the statistical assessment of shape changes between successive vertebrae. A series of 15 homologous landmarks are digitized on the three-dimensional (3D) scans of the CV (C2 to C7) using the software LANDMARK v. 3.0 [67] (Additional file 1: Figure S3). The homologous points capture the vertebral shape in 3D characterizing the morphology of the vertebral centrum and the neural arch. The atlas (first cervical vertebra) is not included in the analysis due to its unique morphology. It lacks specific serial homologies with postatlantal cervicals, and, thus, several landmarks cannot be applied to it.

Analysis and visualization of the geometric morphometric data is performed using the software MORPHOLOGIKA [68]. Two data sets were analyzed: (1) the interspecific data set includes all CV of the four sloths together and (2) the intraneck data sets include all CV of each taxon separately. The interspecific data set allows evaluation of the morphological similarities of the CV across species. The intraneck data sets serve as a basis to identify the morphological modules in each vertebral series. The following procedure was performed on both types of data sets. First, the 3D coordinates of all landmarks were superimposed using a generalized Procrustes superimposition. It removes all the information unrelated to shape [69]. Next, a relative warps analysis was performed to reduce the dimensionality of the dataset and to reveal the similarity relationships among the vertebrae within the cervical vertebral column. The relative warps analysis constructs a morphospace in which shape variation can be quantified. A cluster analysis using the single linkage algorithm in combination with the Euclidian similarity index was performed on the superimposed landmark coordinates. This joins the CV based on minimal distance between them and results in the quantitative establishment of the morphological subunit pattern in the cervical vertebral column.

### *Hox* gene expression and morphological proxies

The somitic *Hox* gene expression pattern in the cervical vertebral column of the mouse was established by a literature survey in Böhmer (2017) [17]. The expression of *Hox* genes of the paralogue groups (PG) 3 to 6 are involved in mediating the development of the cervical vertebral column (e.g., [70]). In particular, the anterior expression limits of *Hox4* and *Hox5* PG are responsible for the regional patterning in the neck and are the focus of the present study. The survey focused on embryonic stages at which the somites are developed along the full anteroposterior body axis and the somitic *Hox* gene expression limits are thought to be well established and stable during further development [16, 19, 23, 58]. Based on the demonstrated correlation between genomic control and phenotypic changes in crocodilians and birds (cf. [16]), the present study of morphological variation of the CV served as a *Hox* gene expression pattern proxy.

Since homeotic transformations induced by mutations of *Hox* genes may be incomplete (e.g., [52, 59, 71, 72] and may result in transitional vertebral identities, the vertebrae were analyzed for the presence of congenital anomalies (such as fusion of vertebrae, left/right asymmetry or rudimentary ribs), following previous studies [15, 26, 73].

## Additional file

Additional file 1: **Figure S1.** Results of cluster analysis for each specimen. A three subunit pattern was revealed in all specimens analyzed. Green, axis; yellow, anterior subunit; red, posterior subunit. A, *C.* cf. *didactylus* 1; B, *C. didactylus* 2; C, *B. variegatus*; D, *B. tridactylus*. **Figure S2.** 3D renderings of all analyzed vertebrae. For each specimen left lateral view above and anterior view below. A, *C.* cf. *didactylus* 1; B, *C. didactylus* 2; C, *B. variegatus*; D, *B. tridactylus*. C1 (atlas) of *B. tridactylus* was not available with the museum specimen. Note that the present analysis involved only postatlantal CV. **Figure S3.** Landmark set used in the 3D geometric morphometric analysis. The numbered 3D landmarks (red points) are shown on a mid-cervical vertebra of *Bradypus tridactylus* (3D model). LM1 = dorsal-anterior edge of vertebral centrum, LM2 = ventral-anterior edge of vertebral centrum, LM3 = ventral-posterior edge of vertebral centrum, LM4 = dorsal-posterior edge of vertebral centrum, LM5 = anteriormost edge of articular facet of postzygapophysis, LM6 = dorsal-posterior edge of articular facet of postzygapophysis, LM7 = point of maximum curvature between postzygapophysis and neural spine, LM8 = posterior edge of neural spine, LM9 = anterior edge of neural spine, LM10 = point of maximum curvature between neural spine and prezygapophysis, LM11 = posteriormost point of articular facet of prezygapophysis, LM12 = dorsal-anterior edge of articular facet of prezygapophysis, LM13 = dorsalmost point of vertebral centrum in anterior view, LM14 = lateralmost point of vertebral centrum in anterior view, LM15 = ventralmost point of vertebral centrum in anterior view. **Table S1.** Percentage of total variance explained and cumulative variance per relative warp (RW). Only the first four RWs are indicated since they explain more than 95% of the total variance. (DOCX 578 kb)
