# Supplementary material for: Homeotic transformations reflect departure from the mammalian ‘rule of seven’ cervical vertebrae in sloths: inferences on the Hox code and morphological modularity of the mammalian neck
Source: BMC Evol Biol. 2018 Jun 7;18:84. doi: 10.1186/s12862-018-1202-5 (PMC5992679; doi:10.1186/s12862-018-1202-5)
Supplement: Supplementary file 1 — Figure S1. Results of cluster analysis for each specimen. A three subunit pattern was revealed in all specimens analyzed. Green, axis; yellow, anterior subunit; red, posterior subunit. A, C. cf. didactylus 1; B, C. didactylus 2; C, B. variegatus; D, B. tridactylus. Figure S2. 3D renderings of all analyzed vertebrae. For each specimen left lateral view above and anterior view below. A, C. cf. didactylus 1; B, C. didactylus 2; C, B. variegatus; D, B. tridactylus. C1 (atlas) of B. tridactylus was not available with the museum specimen. Note that the present analysis involved only postatlantal CV. Figure S3. Landmark set used in the 3D geometric morphometric analysis. The numbered 3D landmarks (red points) are shown on a mid-cervical vertebra of Bradypus tridactylus (3D model). LM1 = dorsal-anterior edge of vertebral centrum, LM2 = ventral-anterior edge of vertebral centrum, LM3 = ventral-posterior edge of vertebral centrum, LM4 = dorsal-posterior edge of vertebral centrum, LM5 = anteriormost edge of articular facet of postzygapophysis, LM6 = dorsal-posterior edge of articular facet of postzygapophysis, LM7 = point of maximum curvature between postzygapophysis and neural spine, LM8 = posterior edge of neural spine, LM9 = anterior edge of neural spine, LM10 = point of maximum curvature between neural spine and prezygapophysis, LM11 = posteriormost point of articular facet of prezygapophysis, LM12 = dorsal-anterior edge of articular facet of prezygapophysis, LM13 = dorsalmost point of vertebral centrum in anterior view, LM14 = lateralmost point of vertebral centrum in anterior view, LM15 = ventralmost point of vertebral centrum in anterior view. Table S1. Percentage of total variance explained and cumulative variance per relative warp (RW). Only the first four RWs are indicated since they explain more than 95% of the total variance. (DOCX 578 kb) [file 12862_2018_1202_MOESM1_ESM.docx]

**SUPPLEMENTARY INFORMATION**

**Böhmer et al. “Homeotic transformations reflect departure from the mammalian ‘rule of seven’ cervical vertebrae in sloths: Inferences on the *Hox* code and morphological modularity of the mammalian neck”**

Content:

Figure S1: Results of cluster analysis for each specimen.

Figure S2: 3D renderings of all analyzed vertebrae.

Figure S3: Landmark set used in the 3D geometric morphometric analysis.

Table S1: Percentage of total variance explained and cumulative variance per relative warp (RW).

**Fig. S1:** Results of cluster analysis for each specimen. A three subunit pattern was revealed in all specimens analyzed. Green, axis; yellow, anterior subunit; red, posterior subunit. A, *C. cf. didactylus* 1; B, *C. didactylus* 2; C, *B. variegatus*; D, *B. tridactylus*.**
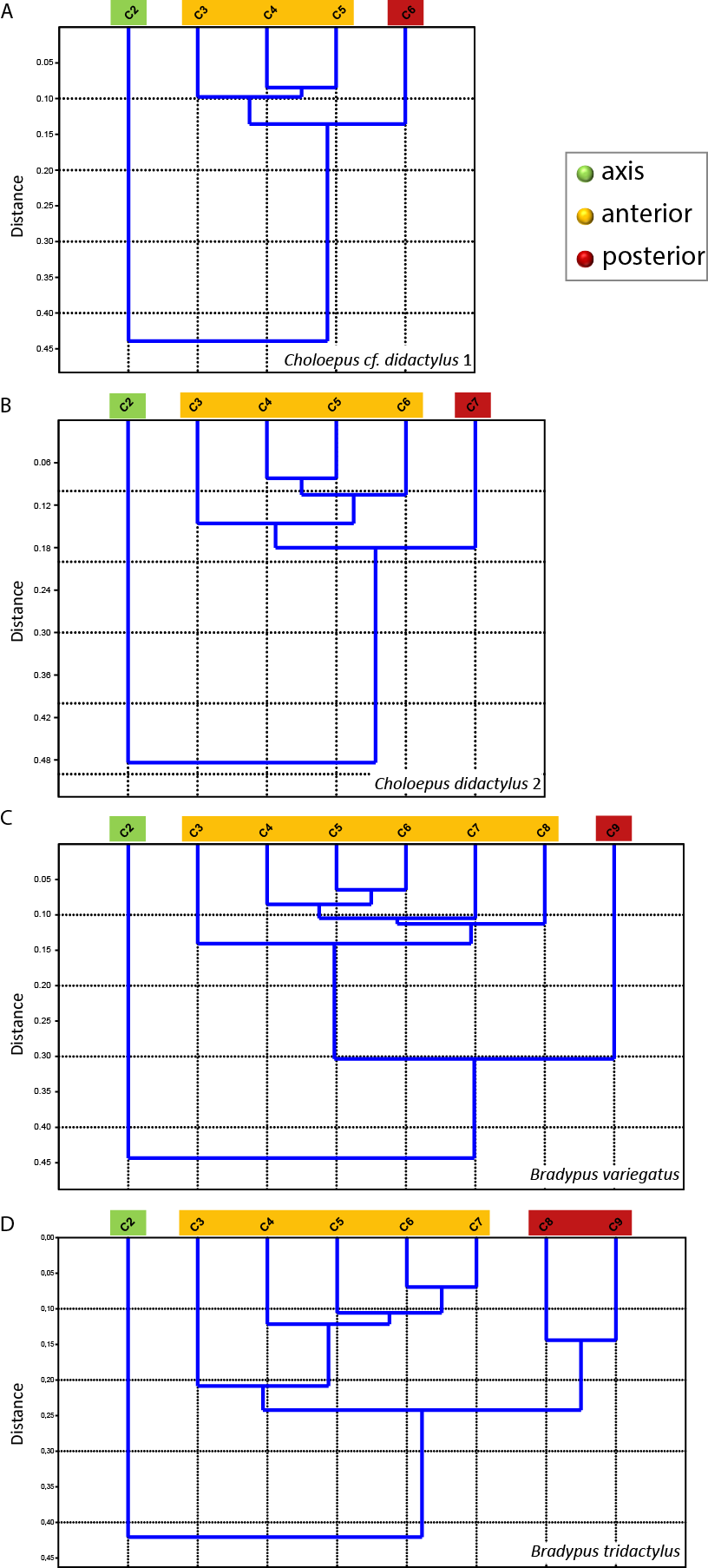
**


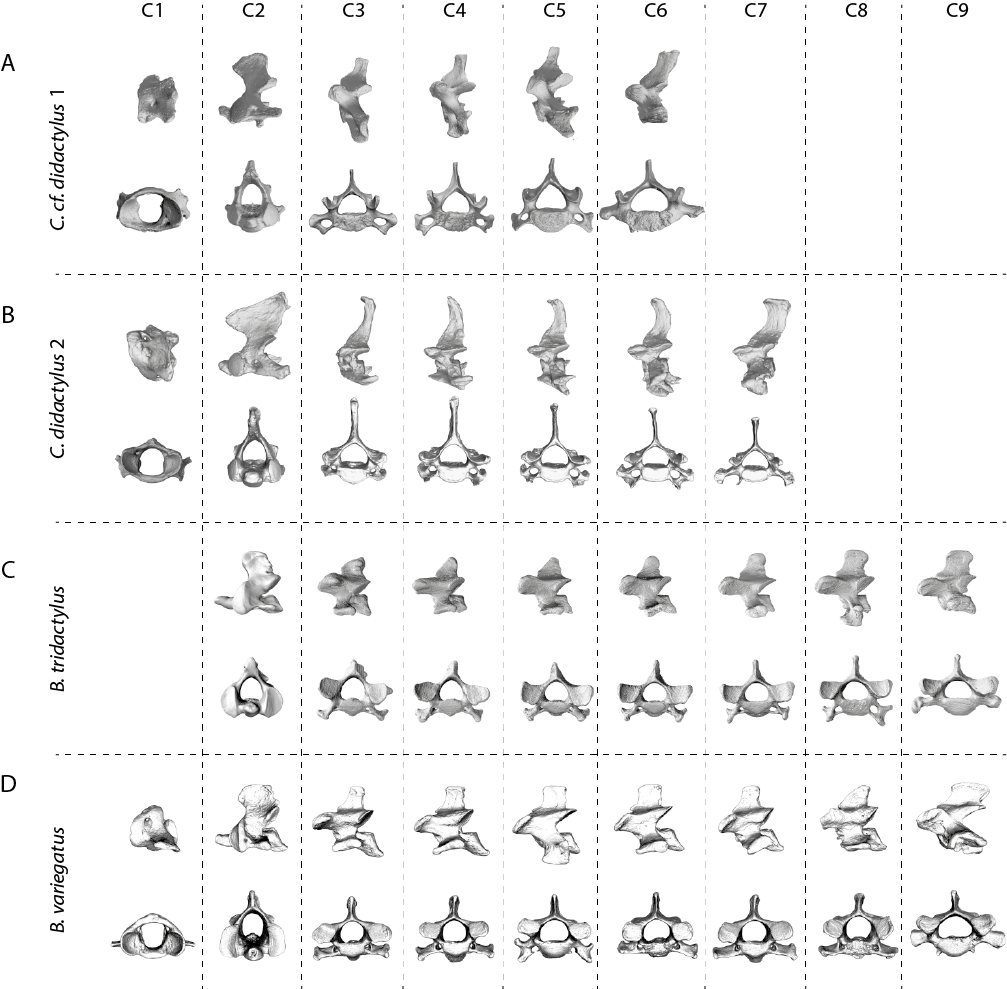


**Fig. S2**: 3D renderings of all analyzed vertebrae. For each specimen left lateral view above and anterior view below. A, *C. cf. didactylus* 1; B, *C. didactylus* 2; C, *B. variegatus*; D, *B. tridactylus*. C1 (atlas) of *B. tridactylus* was not available with the museum specimen. Note that the present analysis involved only postatlantal CV.

**
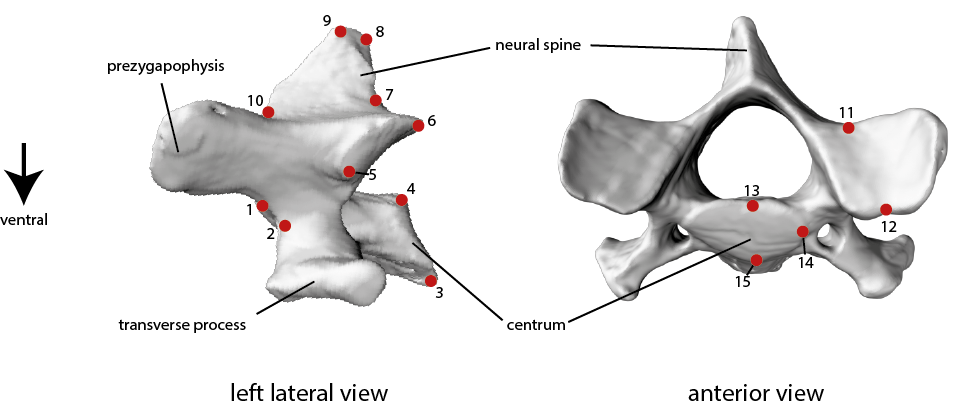
**

**Fig. S3:** Landmark set used in the 3D geometric morphometric analysis. The numbered 3D landmarks (red points) are shown on a mid-cervical vertebra of *Bradypus tridactylus* (3D model). LM1=dorsal-anterior edge of vertebral centrum, LM2=ventral-anterior edge of vertebral centrum, LM3=ventral-posterior edge of vertebral centrum, LM4=dorsal-posterior edge of vertebral centrum, LM5=anteriormost edge of articular facet of postzygapophysis, LM6=dorsal-posterior edge of articular facet of postzygapophysis, LM7=point of maximum curvature between postzygapophysis and neural spine, LM8=posterior edge of neural spine, LM9=anterior edge of neural spine, LM10=point of maximum curvature between neural spine and prezygapophysis, LM11=posteriormost point of articular facet of prezygapophysis, LM12=dorsal-anterior edge of articular facet of prezygapophysis, LM13=dorsalmost point of vertebral centrum in anterior view, LM14=lateralmost point of vertebral centrum in anterior view, LM15=ventralmost point of vertebral centrum in anterior view.

**Table S1**: Percentage of total variance explained and cumulative variance per relative warp (RW). Only the first four RWs are indicated since they explain more than 95% of the total variance.

|  | **Variance [%]** | **Cumulative variance [%]** |
| --- | --- | --- |
| *Choloepus cf. didactylus* 1 | | |
| RW1 | 85.47 | 85.47 |
| RW2 | 9.93 | 95.40 |
| RW3 | 2.81 | 98.21 |
| RW4 | 1.79 | 100.00 |
| *Choloepus didactylus* 2 | | |
| RW1 | 82.66 | 82.66 |
| RW2 | 9.09 | 91.75 |
| RW3 | 4.85 | 96.60 |
| RW4 | 2.09 | 98.69 |
| *Bradypus variegatus* | | |
| RW1 | 59.44 | 59.44 |
| RW2 | 28.98 | 88.42 |
| RW3 | 6.07 | 94.49 |
| RW4 | 2.49 | 96.98 |
| *Bradypus tridactylus* | | |
| RW1 | 55.33 | 55.33 |
| RW2 | 25.73 | 81.06 |
| RW3 | 10.71 | 91.77 |
| RW4 | 4.24 | 96.01 |
